# Does the Animal Model Influence in Vertical Alveolar Distraction? A Systematic Review of the Literature

**DOI:** 10.3390/ani10122347

**Published:** 2020-12-09

**Authors:** Mario García-González, Fernando Muñoz, Antonio González-Cantalapiedra, Mónica López-Peña, Nikola Saulacic

**Affiliations:** 1 Department of Veterinary Clinical Sciences, Faculty of Veterinary, University of Santiago de Compostela, 27002 Lugo, Spain; fernandom.munoz@usc.es (F.M.); antonio.cantalapiedra@usc.es (A.G.-C.); monica.lopez@usc.es (M.L.-P.); 2 Department of Cranio-Maxillofacial Surgery, Faculty of Medicine, University of Bern, 3008 Bern, Switzerland; nikola.saulacic@insel.ch

**Keywords:** animal model, animal experimentation, distraction osteogenesis, biomaterials, alveolar ridge augmentation, VAOD, systematic review

## Abstract

**Simple Summary:**

Vertical alveolar osteogenesis distraction (VAOD) technique appears to provide the best results in terms of vertical bone gain. Before its use in humans, most devices are tested on experimental animals. Currently, there is no consensus on which is the ideal biological animal model for VAOD studies. In this review, it has been found that the animal model influences the results. In addition, the most used, easier to handle, and with less complications was the Mongrel dog. The use of pigs and minipigs, given their difficult handling and poor hygiene, is not recommended.

**Abstract:**

This study is aimed at synthesizing all available evidence on vertical alveolar osteogenesis distraction (VAOD) in animal trials to determine whether the animal model used and its handling influence or not, and find which is the most appropriate animal model for this specific technique. This systematic review has been carried out following the PRISMA statements. Bibliographic sources have been consulted manually by two reviewers. Risk of bias was assessed using a version of the Newcastle-Ottawa-Scale (NOS). The selection criteria established by expert researchers were applied in order to decide which studies should be included in the review. Twenty-six studies met the inclusion criteria and were included in the review. Twenty-four of them had a high quality (score between 7 and 9), two medium quality (score between 4 and 6), and none low quality (score between 1 and 3). The highest possible score was 9 (using the NOS). Six studies complied with all NOS criteria. The animal model has been seen to influence the results, leading to failure in some cases. The most used animal model on VAOD, with fewer complications, was the Mongrel dog. The use of the pig and minipig is not recommended, due to the difficulties in handling and complications encountered.

## 1. Introduction

In oral implantology, one of the most common challenges is the deficiency of mandibular or maxillary alveolar bone height or width. The causes of bone loss include pathological processes such as trauma, periodontal disease, tumor resections, and congenital malformations [1].

Currently, the available techniques that allow the use of long implants without reducing the vertical size of the crown are: augmentation of the floor of the maxillary sinus, the placement of pterygoid implants, and dental nerve transposition [2]. To modify the crown-implant length ratio, other techniques such as guided bone regeneration (GBR), autologous bone grafting, and vertical alveolar distraction can be used [3]. All these techniques increase alveolar ridge height and, thereby, allow the utilization of shorter crowns and large implants.

The biological basis of osteogenic distraction (OD) is called callotasis (etymology: tasis = elongation), which is the progressive elongation of the callus formed around bony portions created by the osteotomy. This elongation process is progressive, allowing for the preservation of the bloodstream. The bone regeneration mechanism is made up of two procedures: histogenesis (elongation of the soft tissues, like blood vessels, nerves, and mucoperiosteum) and osteogenesis (creation of the callus and consequently the generation of new bone) [4].

The OD technique involves the following phases: (A) surgery, (B) latency, (C) distraction, and (D) consolidation. The distraction period includes two different phenomena: the distraction frequency (which is the amount of distraction activations that are performed per day) and the distraction rate (which is the daily amount of bone gained between the basal bone and the transport fragment, measured usually in millimeters) [1].

The vertical alveolar osteogenesis distraction (VAOD) procedure is an effective remedy for soft tissue and bone formation in boneless areas which hinder the rehabilitation with dental implant [1,3]. On the transport segment and the basal bone, a distraction device is placed after executing an osteotomy in the alveolar bone. Periosteum blood flow must be maintained so that the transport segment remains vascularized. Then, the transport segment is submitted to gradual pulling forces to move away from the basal bone. This procedure activates the mechanism which will promote the bone regeneration and the maturity of the distraction callus created [3].

Berhard von Langenbeck carried out, in 1869, the first experimental research on long bone distraction. The fist studies on oral distraction were conducted in 1927 by Rosenthal [4]. In view of the numerous complications that arose, several authors criticized the procedure. In human membranous mandibular bone, the first osteogenic distraction was performed by Snyder et al. in 1973 [5]. Nevertheless, Gabriel Abramowitch Ilizarov developed the full technique in the 1950s, performing numerous experiments in dogs. Thanks to this author, a great advance in the clinical use of the procedure was achieved. Ilizarov also defined the biological basis of the distraction osteogenesis: (1) tension-stress principle influence, (2) bone shape and mass influence, and (3) influence of interaction between mechanical load and blood supply [4].

We can differentiate between two types of distraction gadgets: extraosseous and intraosseous. Depending on their role, there is also a distinction between distractor-implants and distractors [1]. Finally, distractor devices can be classified into horizontal or vertical according to the direction of the regenerated new bone segment.

In a systematic review, Esposito et al. [1] did not have sufficient proof about which was the best procedure. Nevertheless, they informed that VAOD had the best capacity for vertical regeneration among the existing procedures.

Compared to other tissue augmentation treatments, the distraction technique has several advantages [6]:

‑ No donor site morbidity.
‑ Simplest procedure.
‑ Graft resorption less chance.
‑ The quantity gained is more predictable.
‑ The rate of infection and bone resorption is lower
‑ Most of complications are minor.
‑ Possibility of implant placement in the transported fragment.
‑ The consolidation period is shorter, so the treatment has a shorter duration.
‑ When the results are not satisfactory, complementary techniques can be used.

However, it also presents a series of possible disadvantages (Supplementary Table S1) [6,7].

Nonetheless, OD has achieved a certain level of application in maxillofacial and implant surgery as a technique for alveolar bone augmentation. However, there is still no consensus on which is the ideal animal biological model for vertical bone distraction assessment. For the experimental evaluation of alveolar distraction, we need a suitable biological model. This model should offer an adequate amount of bone for the device to be placed on, and subsequently design distraction devices for clinical use in humans. Tests for mandibular alveolar distraction have been developed in small animals, such as rats or rabbits, and in medium and large animals, such as dogs, sheep, minipigs, goats, and primates. Nonhuman primates could represent the ideal biological model for the VAOD research. However, its high cost, the ethical concerns, and difficult handling make it almost not feasible. Currently, the selection of an animal biological model for VAOD is still undefined [8].

This study is aimed at collecting all available evidence on VAOD studies in animals to determine whether the animal model used and its handling influence or not, and find which is the most appropriate animal model for VAOD studies.

## 2. Materials and Methods

This systematic review has been carried out following the PRISMA statements (Supplementary Table S2). Ethics approval was not required for this review. It was performed in the following health science data bases: PubMed, Scopus, and Web of Science (WOS) (limiting the search until December 2019). This search was carried out manually during the month of December 2019 by two reviewers.

The selection criteria incorporated permutations of the following terms: “Alveolar vertical distraction”, “dental implant”, “animal model”, “biological model”, “alveolar ridge”, “vertical alveolar ridge distraction”, “distraction osteogenesis”, “alveolar ridge augmentation”, “alveolar bone loss”, “atrophic jaws”.

PICO methodology. Animal models (P = patients), experimental studies (I = intervention), of different species (C = comparison), used for VAOD (O = result). PICO question: What is the most appropriate animal model to use in VAOD experimental studies?

Eligibility criteria. The studies have been selected according to the following pattern:

1. Experimental studies of alveolar bone distraction aimed at vertical augmentation.
2. Animals used as biological models.
3. Articles in English.

Quality assessment. We used a version of the Newcastle-Ottawa Scale (NOS) to evaluate the quality of the studies with a maximum of 9 stars. Within the analysis, the studies were defined as low quality (1–3), medium quality (4–6), or high quality (7–9). The quality of the studies was assessed in duplicate and independently by two reviewers (M.G.-G and F.M.).

Analysis and extraction of parameters of interest. The studies were evaluated by analyzing the following items: number of animals used (patient number), age, weight, distractor details, number of distractors used, number of distraction segments, location of the distractor (jaw/maxilla and segment site), distraction succession (phase of latency, phase of activation and phase of consolidation), rate and frequency of distraction, vertical bone gain obtained at the end of the study, specification of the quantification method analysis, and description of the complications observed.

Regarding the studies in which complications were observed, they were classified as minor and major [9]. In addition, the phase in which they were observed was detailed [10].

## 3. Results

**Figure 1:**
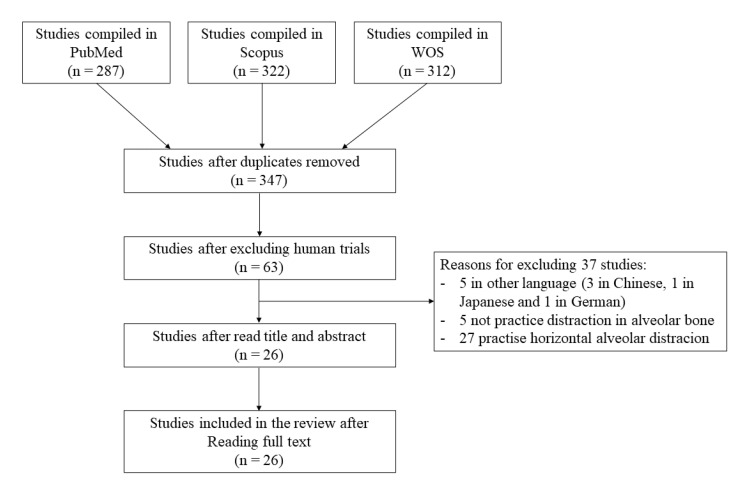
Flow chart of the selected studies.

The initial search generated 921 articles. Figure 1 shows the flow chart of the selected articles submitted to the review process. After the exclusion of duplicates and studies in humans, there was a remaining total of 63 articles. Based on abstracts and titles, 37 studies were removed after applying study criteria; 5 studies were written in a language other than English (3 in Chinese, 1 in Japanese, and 1 in German), 5 were eliminated because they did not practice oral distraction in the alveolar bone, and 27 studies were removed because horizontal distraction was performed. After full text reading, 26 studies [8,11,12,13,14,15,16,17,18,19,20,21,22,23,24,25,26,27,28,29,30,31,32,33,34] complying with the inclusion rules were selected for the review. Twenty studies were in dogs (with a total of 145 animals and 223 placed devices), 2 in rabbits (72 animals and 72 devices), 2 in sheep (18 animals and 26 devices), 1 in minipigs (3 animals and 3 devices), and 1 in baboons (3 animals and 3 devices).

Based on this review, 24 articles were rated as high quality, 2 as medium quality, and 0 as low quality. Six studies obtained a maximum score of 9 stars (Table 1).

All animals used were adults and skeletally mature. The average age by species is 18 months in dogs and 13 months in sheep. The most commonly used breed was Mongrel in dogs, New Zealand in rabbits, Gottingen in minipigs, and Papio Anubis in non-human primates. The average weight was 17.3 kg in dogs (ranging from 9 to 40 kg), 4.1 kg in rabbits (ranging from 4.15 to 0.55 kg), and 45 in sheep.

The 26 articles selected involved 241 patients with 327 distractors; 158 patients had been treated with an extraosseous and 83 with an intraosseous device (Table 2).

Indication and location for distraction. In 23 studies (217 patients), the jaw was used for distraction and in 3 the maxilla (24 patients). The most common location for distraction was the posterior region of the left jaw in all species (82 patients), followed by the posterior region of the right jaw (59 patients); 220 patients had unilateral and 21 bilateral oral distraction (Supplementary Table S3).

Device details. Thirteen different types of commercial distraction devices and other prototypes were found:

‑ 8 intraosseous devices: 3i (Standard threaded implant, 3i System, Implant Innovations, West Palm Beach, FL, USA), DL-system (Dep. of Oral & Maxillofacial Surgery, Fourth Military Medical University, Shaanxi, China), AW (Alveo-Wider; Okada Medical Supply, Tokyo, Japan), DK KXSLD (Dikang Biomedical Co., Ltd., Chengdu, China), STATA 4.0 (STATA Corp, College Station, TX, USA), OMS (Oral & Maxillofacial Surgery, School of Stomatology, The 4th Military Medical Univ, Xi’an, China), ABDUL (Dentium Co Ltd., Seoul, Korea & Osstem Co Ltd., Seoul, Korea), and DID (DID-Trade Inc., Klagenfurt, Austria).
‑ 5 extraosseous devices: KLS Martin (Tuttlingen, Germany), OPD (U-shaped body, Synthes Maxillofacial, Paoli, PA, USA), KLS Martin (Jacksonville, FL, USA), TRACK 1.0 mm-System (Gebrüder Martin GmbH & Co. KG, Tuttlingen, Germany), and Impladent SL (Barcelona, Spain).

The most commonly used distractor device was OPD, which was employed in 2 studies and treated 72 patients [33,34]. The same prototype of Impladent SL (Barcelona) was used in 2 studies to treat a total of 11 patients [8,11]. The rest of devices were used in just 1 study.

The total number of movable segments were 278. One or more devices may have been placed per segment.

VAOD protocol. A latency period of 7 days was by most of the studies, with a mean of 6.13, ranging from 0 [15] to 8 days [12]. In the distraction period, there were multiple sequences in terms of frequency and rate. This was the period with most discrepancies. However, in none of the studies was performed the daily augmentation of over 1 mm. Ten days was the distraction period in most of the studies, with an average of 9.15 (ranging from 5 to 28) days. The mean distraction rate was 0.8 (0.25 to 1 mm) per day. The distraction frequency varied between 1 and 2 times per day. In one study, the frequency was 1 time every two days [13]. The consolidation period average was 9.93 weeks (ranging from 0 to 50 weeks). Zhan et al. [15] did not have a consolidation period (0 weeks). Oda et al. [18] applied the longest consolidation period (50 weeks). Table 3 shows in detail the characteristics of the latency, distraction, and consolidation phases of the studies included in the review.

Vertical bone gain. The main gain achieved in these studies was 6.13 mm (ranging from 2.72 to 8.96 mm). The average gain for extraosseous distractors was 6.26 mm and 6.02 in the case of intraosseous distractors. The study with the highest gain was that of Xie et al. [30], who recorded a gain of 8.96 mm, using an intraosseous distractor (STATA 4.0) (STATA Corp, College Station, TX, USA), whereas the lowest gain (2.72 mm) was obtained by Esposito et al. [12], using an intraosseous prototype. For extraosseous devices, Block et al. [24] obtained the highest bone gain (8.85 mm) using a prototype, while the lowest (3.25 mm) was obtained by Terbish et al. [14], using a prototype device.

Quantification methods. The quantification methods used whereas follows: histomorphometry (21 studies), X-rays (17 studies), microtomography (5 studies), immunohistochemistry (1 study), and fluorescence microscopy (1 study).

Complications and treatment. Complications were found in 12 studies, whereas 11 studies claimed to have had no complications; 3 of them did not supply information.

Out of 241 patients, 24 (9.95%) exhibited complications; 11 of them (4.56% of the total) were major and 13 (5.1% of the total) minor complications. By order of frequency, the minor complications were infection (5 patients, 20.83% out of complications), edema (5, 20.83%), dehiscence (2, 8.32%), and alveolar arterial hemorrhage (1, 4.16%). Using the same criteria, the major complications were: device removal due to a moderate infection (6, 25%), support plate deviation (2, 8.32%), intra-surgical death (1, 4.16%), mandibular fracture (1, 4.16%), and severe diarrhea and death (1, 4.16%).

In 3.31% of the total patients, the distractor was affected due to moderate infection and death of animals, which had to be removed from the study.

Table 4 shows the complications and treatment classified according to the protocol phase.

## 4. Discussion

This review focused on a total of 26 studies that evaluated VAOD. So far, there is no consensus about which is the ideal animal for vertical alveolar distraction tests. In this review, the most commonly used animal, with the least complications, was the dog (20 out of 26 studies; 145 out of 241 animals; 140 out of 327 devices). Among the breeds, Mongrel was the most used (11 out of 20 studies).

Bone regeneration in OD has been related to the age of the patient; faster in young patients, and slower in older ones. In a study performed in rats aged between 4 and 24 months, statistically significant differences were observed in the radiodensity of distracted segment of tibiae; 95% of mineralized bone was observed in 4 month-old rats, whereas in 24 month-old rats, up to 36% of mineralized bone was observed [35]. All the animals included in this review were skeletally mature (average of 18 months in dogs and 13 months in sheep), suggesting that the results were not affected by the patient’s age.

Although the procedure of VAOD has been used for over 25 years, there is still controversy over the ideal VAOD protocol. However, the clinical phases of the process (osteotomy, latency, distraction, and consolidation) have remained the same. Most of the studies in this review used a latency phase of 7 days, with a mean of 6.13 (ranging from 0 to 8) days, with similar results. Altug et al. [16] compared two different latency periods (1 and 7 days) in rabbits, not observing differences in the characteristics and quantity of the new bone. In human studies, no significant differences were observed. In a set of 3278 dossiers of clinical cases of cranio-facial distraction, Mofid et al. [36] did not find any significant differences between clinicians that established a latency period and those who did not, in terms of parameters such as absence of fibrous union (0.4% vs. 0.25%) or premature consolidation (2% vs. 0.76% of cases). However, a latency period of 4–7 days is advisable in alveolar OD with the objective of avoiding premature exposure of bone to the oral environment [37]. This issue may be even more important when we use an animal model.

The period between the initial and the final activation of the distractor is called distraction period. There are multiple options for rate and distraction frequency. Most authors agree on a maximum of 1 mm per day. In this review, the rate oscillated between 0.25 and 1 mm, with a mean of 0.8 mm per day. There are no previous reviews of studies on animals. In humans, a rate ranging between 0.375 and 1 mm [3] or between 0.44 to 0.98 mm [10] per day was found.

Regarding the frequency of distraction, this delicate phase needs to be performed by specially trained staff. In this study, the ranges varied mostly from one to two times per day. In one study, distraction was used once every two days [13]. When distraction is applied more than 2 times a day it can complicate the procedure [3] and may be the reason for applying a low rate of distraction in animals.

Osteogenic distraction is a technique with the capacity of producing an increase in the height of the alveolar bone that may vary between 4 and 15 mm, with a mean of 9.9 mm [38]. The mean gain obtained by VAOD in this review was 6.13 mm, being greater with extraosseous distraction devices than with intraosseous ones (6.26 vs. 6.02 mm). A limitation of the present review was that the vertical gain obtained by VAOD was not directly compared with other surgical techniques for bone augmentation like xenogenic (5.2 ± 0.79 mm) or alloplastic materials, autogenous bone grafts, or allografts [39]. Nevertheless, since the height gained with these techniques was lower (e.g. 5.2 ± 0.79 mm for xenogenic), the VAOD technique may be considered as most suitable when the objective is the vertical bone gain [1,3].

In the consolidation period, an average of 9.93 weeks was observed (ranging between 0 and 50 weeks). Many discrepancies were found in comparison with studies in humans, with a mean of 12.05 [3], 11.22 [10], and 11.83 weeks [40]. To observe the fusion of the basal bone with the distracted segment, a minimum of 10 weeks of consolidation was needed [3]. Shorter consolidation periods used in animals may be explained by the faster metabolism compared to humans.

Multiple ways to classify complications were studied. In this review, we used a modification of the classification carried out by Enislidis et al. [9] and Saulacic et al. [10]. These authors classified the complications into major and minor, and based on when these complications occurred. We found complications in 12 studies (24 patients, 9.95% of the total). Eleven patients (4.56% of the total) showed major and minor complications in 13 studies (5.39%). Out of the patients with major complications, 9 had to be discarded (3.73% of failures). Similar failure rates were observed in human studies, with 3.44% of failures [3]. Other major complications were solved with the relocation of the distraction device.

The maintenance of an appropriate level of oral hygiene in animals is sometimes a great challenge. Therefore, surgical sites in the mouth of animals are more likely to get infected, thus increasing the failure rate. Management of pigs and minipigs may be especially difficult, due to the chewing habits, affecting the failure rate

According to other studies, the most common minor complication is dehiscence [9,38]. It is also advisable to reduce the frequency and the rate of distraction if the dehiscence is considerable and persists [3]. For minor complications, such as inflammations, dehiscence, and infections, conservative treatment is advised. Minor complications usually have a simple solution, which does not interfere with the results of the technique.

## 5. Conclusions

The animal model used in VAOD studies has a marked influence on the results. Within the limitations of the present study, the animal model mostly used and recommendable in VAOD, which shows the least complication was the Mongrel dog. Given the difficulties in handling along with the complications encountered, the use of the pig and minipig as a model for VAOD is not recommended. The possibility to achieve an adequate level of oral hygiene and to reduce the rate and rhythm of activation should be further considered when selecting a suitable animal model for VAOD.

## Figures and Tables

**Table 1:** Score by studies using NOS Scale.

Author	Year	Specie	Stars	Quality
Cano [11]	2006	Dog	9	High
Esposito [12]	2017	Dog	9	High
Li [13]	2014	Dog	9	High
Terbish [14]	2015	Dog	9	High
Zhan [15]	2018	Dog	9	High
Shao [21]	2013	Dog	9	High
Altug [16]	2011	Rabbit	8	High
Faber [17]	2005	Dog	8	High
Oda [19]	2000	Dog	8	High
Oda [18]	1999	Dog	8	High
Rachmiel [20]	2004	Sheep	8	High
Yi [22]	2009	Dog	8	High
Zhao [23]	2009	Dog	8	High
Block [24]	1998	Dog	8	High
Block [25]	1996	Dog	8	High
Block [26]	2000	Dog	8	High
Demetoglu [27]	2017	Dog	8	High
Martínez-González [8]	2005	Minipig	8	High
Martínez-González [8]	2005	Dog	8	High
Perry [28]	2006	Dog	8	High
Takeuchi [29]	2010	Dog	8	High
Xie [30]	2012	Dog	7	High
Boyne [31]	2004	Baboon	7	High
Gaggl [32]	2000	Sheep	7	High
Bayar [33]	2012	Rabbit	6	Medium
Hwang [34]	2004	Dog	5	Medium

**Table 2:** Individual parameters of each study (P = prototype)**.**

Author	Year	Distractor Design	Animal	Breed	Patient Number	Age (Months)	Weight (Kg)	N° Distractors	N° Segments	Extraosseous	Intraosseous
Terbish [14]	2015	P	Dog	Beagle	16	16 to 18	15 to 16	16	16	16	/
Oda [18]	1999	3i	Dog	Mongrel	5	Adult	15 to 17	10	5	/	10
Takeuchi [29]	2010	TRACK	Dog	Beagle	11	12 to 24	10	11	11	11	/
Zhao [23]	2009	DL	Dog	Mongrel	6	18	/	12	6	/	12
Block [24]	1998	P	Dog	Mongrel	4	Adult	/	4	4	4	/
Cano [11]	2006	Impladent SL	Dog	Beagle	5	24	10 to 15	4	5	4	/
Faber [17]	2005	P	Dog	Mongrel	5	Adult	11 to 16.4	10	5	10	/
Perry [28]	2005	P	Dog	American foxhound	5	Adult	25 to 30	5	5	/	5
Demetoglu [27]	2017	P	Dog	Beagle	10	Adult	35 to 40	20	20	/	20
Oda [19]	2000	P	Dog	Mongrel	6	Adult	17 to 20	12	6	6	/
Block [25]	1996	P	Dog	Mongrel	4	Adult	/	4	4	4	/
Li [13]	2014	DK KXSLD	Dog	Mongrel	9	24	20	18	18	/	18
Block [26]	2000	P	Dog	Mongrel	8	Adult	/	8	8	/	8
Xie [30]	2012	STATA 4.0	Dog	Mongrel	18	Adult	21 to 26	36	18	/	36
Shao [21]	2013	OMS	Dog	Mongrel	6	24	/	18	12	/	12
Hwang [34]	2004	ABDUL	Dog	Beagle	4	Adult	/	4	4	/	4
Yi [22]	2009	P	Dog	Mongrel	4	Adult	9 to 10	8	8	8	/
Esposito [12]	2017	P	Dog	Beagle	4	19 to 27	12.8	16	12	/	16
Zhan [15]	2018	P	Dog	Beagle	12	12 to 15	7 to 10	4	12	4	/
Rachmiel [20]	2004	KLS Martin	Sheep	/	10	12	45	10	10	10	/
Gaggl [32]	2000	DID	Sheep	/	8	36	/	16	8	/	16
Martínez-González [8]	2005	Impladent SL	Dog	Beagle	3	Adult	/	3	3	3	/
Martínez-González [8]	2005	Impladent SL	Minipig	Göttingen	3	Adult	/	3	3	3	/
Boyne [31]	2004	KLS Martin	Monkey	Papio anubis baboons	3	Adult	/	3	3	3	/
Altug [16]	2011	OPD	Rabbit	New Zealand	36	Adult	4.15 ± 0.55	36	36	36	/
Bayar [33]	2012	OPD	Rabbit	New Zealand	36	Adult	/	36	36	36	/

**Table 3:** Distraction protocol details of each study.

Author	Year	Distractor Detail	Animal	Latency Days	Distraction Days	Consolidation Weeks	Rate/Frequency	Gain (mm)
Terbish [14]	2015	P	Dog	7	10	2 to 6	0.4 mm/twice daily	3.25 ± 0.95
Oda [18]	1999	3i	Dog	7	6	50	0.9 mm/day	3.98 ± 0.39
Takeuchi [29]	2010	TRACK	Dog	7	6	8	0.9 mm/day	-
Zhao [23]	2009	DL	Dog	7	8	12	1 mm/day	7.25 ± 0.40
Block [24]	1998	P	Dog	7	10	10	0.5 mm/twice daily	8.85
Cano [11]	2006	Impladent SL	Dog	7	5	4 and 8	1 mm/day	5.49 ± 0.9
Faber [17]	2005	P	Dog	7	9	0 to 14	0.65 mm/day	5.45 ± 2.01
Perry [28]	2005	P	Dog	7	10	12	0.5 mm/twice daily	-
Demetoglu [27]	2017	P	Dog	7	10	12	1 mm/day	-
Oda [19]	2000	P	Dog	7	7	8 or 12	1 mm/day	6.51
Block [25]	1996	P	Dog	7	10	10	0.5 mm/twice daily	-
Li [13]	2014	DK KXSLD	Dog	5	12	4, 8 and 12	1 mm/2 days	5.71
Block [26]	2000	P	Dog	7	10	10	0.5 mm/twice daily	8.8 ± 1
Xie [30]	2012	STATA 4.0	Dog	7	-	12 and 24	-	8.96 ± 1.89
Shao [21]	2013	OMS	Dog	5	12	4, 8 and 12	1 mm/2 days	5.8 ± 0.2
Hwang [34]	2004	ABDUL	Dog	-	-	-	-	-
Yi [22]	2009	P	Dog	7	5	6	0.5 mm/twice daily	-
Esposito [12]	2017	P	Dog	8	8	10	0.75 mm/day	2.72
Zhan [15]	2018	P	Dog	0	28	0	0.5 mm/2 days	-
Rachmiel [20]	2004	KLS Martin	Sheep	5	24	12	0.5 mm/day	-
Gaggl [32]	2000	DID	Sheep	7	8	4, 8, 12 and 24	0.5 mm/day	-
Martínez-González [8]	2005	Impladent SL	Dog	7	5	2	1 mm/day	-
Martínez-González [8]	2005	Impladent SL	Minipig	7	5	2	1 mm/day	-
Boyne [31]	2004	KLS Martin	Baboon	7	10	20	1 mm/day	-
Altug [16]	2011	OPD	Rabbit	1 and 7	10	2, 4 and 8	0.25 mm/twice daily	-
Bayar [33]	2012	OPD	Rabbit	7	10	2, 4, and 8	0.25 mm/twice daily	-

**Table 4:** Complications and treatments of the selected studies.

Author	Year	No. Patients	Complications	Affected Patients	Period	Major/Minor	Treatment
Terbish [14]	2015	16	Infection	2 (12.5%)	Phase 3. Distraction	Minor	Conservative
Oda [18]	1999	5	Dehiscence	2 (40%)	Phase 3. Distraction	Minor	Conservative
			Infection	2 (40%)	Phase 4. Consolidation	Minor	Conservative
Takeuchi [29]	2010	11	No				
Zhao [23]	2009	6	Several diarrhea	1 (16.67%)	Phase 2. Latency	Major	Death
Block [24]	1998	4	Alveolar artery cut	1 (25%)	Phase 1. Surgery	Minor	Suture
Cano [11]	2006	5	Moderate Infection	2 (40%)	Phase 2. Latency	Major	Device removal
Faber [17]	2005	5	Moderate edema	5 (100%)	Phase 2. Latency	Minor	Conservative
Perry [28]	2005	5	No				
Demetoglu [27]	2017	10	Intra-surgery death	1 (10%)	Phase 1. Surgery	Major	Death
			Infection	1 (10%)	Phase 4. Consolidation	Minor	Conservative
Oda [19]	2000	6	Support plate deviation	2 (33.34%)	Phase 3. Distraction	Major	Screw recolocation
Block [25]	1996	4	No				
Li [13]	2014	9	No				
Block [26]	2000	8	No				
Xie [30]	2012	18	No				
Shao [21]	2013	6	No				
Hwang [34]	2004	4	*	*	*	*	*
Yi [22]	2009	4	No				
Esposito [12]	2017	4	Mandibular fracture	1 (25%)	Phase 4. Consolidation	Major	Euthanasia
Zhan [15]	2018	12	No				
Rachmiel [20]	2004	10	No				
Gaggl [32]	2000	8	*	*	*	*	*
Martínez-González [8]	2005	3	Moderate Infection	1 (33.34%)	Phase 3. Distraction	Major	Device removal
Martínez-González [8]	2005	3	Moderate Infection	3 (100%)	Phase 2. Latency	Major	Device removal
Boyne [31]	2004	3	*	*	*	*	*
Altug [16]	2011	36	No				
Bayar [33]	2012	36	*	*	*	*	*

* No mention any about complications.

## Supplementary Materials

The following are available online at https://www.mdpi.com/2076-2615/10/12/2347/s1, Table S1: Most common complications in VAOD, prevention and treatment. Table S2: PRISMA 2009 Checklist. Table S3: Indication and location for distraction.
